# Monitoring skeletal muscle oxygen saturation kinetics during graded exercise testing in NCAA division I female rowers

**DOI:** 10.3389/fphys.2025.1538465

**Published:** 2025-02-17

**Authors:** Drake A. Eserhaut, Joseph M. DeLeo, Jessica A. Provost, Kathryn E. Ackerman, Andrew C. Fry

**Affiliations:** ^1^ Jayhawk Athletic Performance Laboratory – Wu Tsai Human Performance Alliance, University of Kansas, Lawrence, KS, United States; ^2^ Female Athlete Program - Wu Tsai Human Performance Alliance, Boston Children’s Hospital, Boston, MA, United States

**Keywords:** near-infrared spectroscopy, blood lactate, physiological thresholds, longitudinal monitoring, performance testing

## Abstract

**Purpose:**

The purpose of this study was to analyze changes in skeletal muscle oxygen saturation (SmO_2_) kinetics during exercise in female rowers both acutely and longitudinally in relation to blood lactate (BLa). We also aimed to determine the agreement and statistical equivalence between physiological thresholds derived from SmO_2_ and BLa kinetics.

**Methods:**

Twenty-three female NCAA Division I rowers were tested throughout the 2023–2024 academic year. Of these, 11 athletes completed at least two near-infrared spectroscopy (NIRS)-equipped GXTs, with physiological data analyzed for longitudinal changes. A 7x4-min discontinuous GXT protocol was performed by all athletes. First and second SmO_2_ breakpoints (SmO_2_BP1 and SmO_2_BP2) were estimated via piecewise linear regression modeling, and BLa thresholds (LT_1_ and LT_2_) were calculated using ADAPT software. Paired-samples t-tests assessed differences, and equivalence was tested using two one-sided tests (TOST). Agreement was determined using Bland-Altman analysis yielding mean differences (MD) and 95% limits of agreement (LoA). Intraclass correlation coefficients (ICC_2,1_) were also calculated.

**Results:**

No difference was found between SmO_2_BP2 and LT_2_ (MD = −5.76W [95% LoA = −38.52 to 22.25W], p = 0.134), moderate-to-good levels of agreement (ICC_2,1_ = 0.67 [95% CI: 0.36–0.85], p < 0.001), and no statistical equivalence (p = 0.117). This was not the case for SmO_2_BP1 and LT_1_, with NIRS significantly underestimating LT_1_ (MD = −8.14W [95% LoA = −38.90 to 27.37W], p = 0.026), poor-to-moderate agreement (ICC_2,1_ = 0.24 [95% CI: −0.13–0.58], p = 0.10), and no statistical equivalence (p = 0.487). Additionally, SmO_2_ recovery kinetics (SmO_2_resat) during 1-min rest intervals increased in response to graded increases in exercise intensity (p < 0.001, η^2^_p_ = 0.71), with higher intensities appearing to blunt this effect (step 6 – step 7: MD = −0.16%⋅s^-1^, p = 0.69). No statistically significant changes were observed in LT’s or SmO_2_BP’s throughout the 2023–2024 season.

**Conclusion:**

In female collegiate rowers, NIRS may be a tool that compliments BLa testing when determining the second lactate threshold (i.e., LT_2_). However, significant inter-individual variablility exists between SmO_2_BP2 and LT2 paired with a lack of statistical equivalence suggest the two are not interchangeable. While not a standalone replacement, if used in combination with traditional BLa testing methods NIRS may be a complimentary tool that helps inform individual athlete training zone prescription.

## Introduction

The rowing stroke is a cyclic movement that can be separated into two distinct phases: the drive (propulsive) phase and the recovery phase. During the recovery phase, athletes extend their arms, rockover from their hips, then begin to fully flex the knees and hips as they glide forward on the seat with the heels slightly rising as they arrive at the catch position. During the drive phase, rowers generate propulsive force by sequentially using the legs, trunk, and arms to overcome the drag from the water and rowing shell (Nugent et al., 2020) or the resistance from the rowing ergometer. Once the rower completes the stroke, their legs are fully extended, trunk angle is typically at 20–25°, and the handle is at the furthest point towards the bow (Kleshnev, 2016). Elite rowers’ power production during the rowing stroke has been reported as deriving 43% from the legs, 36% from the trunk, and 21% from the arms (Kleshnev, 2016). Yet, in order to better optimize training for the standard 2,000-m Olympic race distance (2KOrd), we should also consider specific muscle and energy system contributions to the standard 2,000-m rowing ergometer performance (2KErg).

Several studies have investigated on-water (Fleming et al., 2014) and ergometer (Janshen et al., 2009; Turpin et al., 2011; Pollock et al., 2012; Wilson et al., 1988) rowing to quantify EMG activity of various muscle groups in the legs, trunk, and arms to determine their contribution to the drive phase of the rowing stroke. Collectively across these various studies, the surface EMG studies of the lower extremities show that the drive phase is dominated by the following muscles: vastus lateralis (VL), vastus medialis (VM), gluteus maximus (GM), rectus femoris (RF), biceps femoris (BF), tibialis anterior (TibA), and gastrocnemius (Gastroc) (Fleming et al., 2014; Janshen et al., 2009; Turpin et al., 2011; Pollock et al., 2012; Wilson et al., 1988; Soper and Hume, 2004). The VL, GM, BF, and Gastroc reach peak muscle EMG activity just prior to and during peak force at the handle (Wilson et al., 1988), which is at 50% of the drive phase (Janshen et al., 2009; Wilson et al., 1988). It has been suggested that the VL contributes more to the drive phase during ergometer rowing while the VM is recruited more heavily during on-water rowing (Fleming et al., 2014). Furthermore, there is evidence of significant asymmetrical lower extremity muscle activity in sweep rowers (Janshen et al., 2009). Janshen et al. found that the VL and RF EMG activity of the inside leg of sweep rowers were 25% and 32% higher, respectively, than the outside leg (Janshen et al., 2009). Van der Zwaard et al. studied 18 elite Dutch rowers to determine the relationship of various skeletal muscle physiology characteristics and 2KErg performance (van der Zwaard et al., 2018). Van der Zwaard and colleagues evaluated the muscle volume, cross-sectional area, fascicle length, and pennation angle of the medial VL. They found that the medial VL volume explained 86% of the variance of 2KErg. Furthermore, the medial VL volume had the strongest relationship (r = 0.92, p = < 0.01) to 2KErg when compared to muscle cross-sectional area (r = 0.83, p = < 0.01), fascicle length (r = 0.66, p = < 0.05) and pennation angle (r = −0.06, p = > 0.05) (van der Zwaard et al., 2018). Taken together, these studies emphasize that the VL is one of the primary muscles for creating power during the drive phase of the rowing stroke. Therefore, this indicates that the VL is a critical muscle to monitor and continually evaluate in the training and testing of rowers to optimize performance.

Rowers create the power to propel the boat across the 2KOrd with energy system contributions attributed 70%–77% to aerobic metabolism and 23%–30% to anaerobic metabolism (Martin and Tomescu, 2017; Hagerman, 1984). The distribution of exercise metabolism, across the 2KOrd, emphasizes the need for rowers to develop the physical attributes of endurance and maximal strength to achieve peak performance. Historically, two of the most popular methods to assess rowers’ exercise metabolism and determine training zone prescription during graded exercise tests (GXT) are blood lactate (BLa) (Kindermann et al., 1979; Mader, 1976; Faude et al., 2009) and gas exchange (Bhambhani and Singh, 1985; Wasserman and McIlroy, 1964; Wasserman et al., 1973). While these methods are considered the commonly used for physiological testing and training zone prescription, they can be difficult to implement due to three primary constraints: 1) limited access to laboratory equipment; 2) lack of staff with the knowledge and expertise necessary to conduct and analyze results of the physiological testing; and 3) cost–both time and financial.

Recently, near infrared spectroscopy (NIRS) has been investigated as a non-invasive technology to potentially identify the two breakpoints associated with the first and second BLa thresholds (LT_1_ and LT_2_) (Batterson et al., 2023; Iannetta et al., 2017; Possamai et al., 2024; Snyder and Parmenter, 2009; Turnes et al., 2019). The use of NIRS technology directly addresses two of three primary constraints to BLa testing: a relatively low-cost tool that can be applied directly on-water in the rowing shell or on-land on the rowing ergometer, therefore bypassing the need for laboratory access. The identification of the two NIRS breakpoints is accomplished by placing a NIRS device on the primary muscle(s) that would be used during the exercise activity; in this case the VL of rowers. Once the NIRS device is positioned, the infrared light (700–900 nm) can penetrate several millimeters of tissue reaching hemoglobin, myoglobin, and cytochrome oxidase chromophores within the skeletal muscle tissue of interest. Hemoglobin and myoglobin’s ability to absorb and scatter light is dependent upon whether it is bound to oxygen (O_2_) or not (Barstow, 2019). Therefore, when the infrared light reaches one of these chromophores, the light signal is then sent back to the NIRS device, providing real-time data on local muscle oxygen supply and demand during exercise.

The identification and improvement of power at LT_1_ and LT_2_ has important performance implications for endurance sport (Mader, 1976; Ingham et al., 2002; Mader et al., 1978). The power at 4 mmol L^-1^, a fixed blood lactate concentration (FBLC), is often used as LT_2_ has been found to be a key determinant of 2KErg performance (Ingham et al., 2002; Homer, 2014). Mark Homer, former British Rowing physiologist, found that the power at a FBLC of 4 mmol L^-1^ was the only significant determinant of 2KErg for males and females at the U23 and Senior competition levels (Homer, 2014). Therefore, the monitoring and evaluation of power at LT_1_ and LT_2_ should be considered an integral part of a rowing training program. Several studies have investigated the capability of NIRS to identify LT_1_ and LT_2_ with evidence to support (Batterson et al., 2023; Iannetta et al., 2017; Snyder and Parmenter, 2009) and contradict its efficacy (Lanigan, 2022; Passamai et al., 2024; Turnes et al., 2019). Turnes et al. investigated the relationship between a single breakpoint in deoxygenated hemoglobin (HHb|BP), using the PortaMon NIRS device, and the anaerobic threshold (AnT) during an incremental test and its relationship to 2KErg. For clarity, HHb is different than other NIRS terms such as tissue saturation index (TSI) and skeletal muscle oxygen saturation (SmO_2_), as the two later variables represent hemoglobin that is *actively bound to oxygen*, whereas HHb reflects the total volume of hemoglobin *unbound to oxygen.* Further, Turnes and colleagues defined the AnT as a FBLC of 3.5 mmol L^-1^ determined by linear interpolation, and HHb|BP was determined via a piecewise linear regression, with the breakpoint corresponding to the estimated intercept of the two linear regression lines (Turnes et al., 2019). After estimation, HHb|BP was visually identified by two independent investigators as the inflection point between the two linear regression lines, followed by a notable plateau in HHb. They found a significant correlation between (HHb|BP) and AnT (r = 0.63, p = 0.02), with no difference between mean power at (HHb|BP) and AnT (236 vs 234 W, respectively). However, there was high typical error of estimate (10.7%) and wide 95% limits of agreement (−54.1 to 50.6 W); such high variability should caution coaches and sport scientists about using this technology with rowers (Turnes et al., 2019).

In a separate study, Possamai et al. sought to investigate the (HHb|BP) along with TSI breakpoints (TSI-BP) in four scenarios during discontinuous (3-min stages, HHb|BP_3_, TSI-BP_3_), continuous (1-min stages, HHb|BP_1_, TSI-BP_1_), and constant workload tests to determine maximal lactate steady state (MLSS) and rowing distances of 500, 1,000, 2000, and 6000-m to determine critical power (CP). Possamai et al. used the same methodology as Turnes et al. to determine HHb|BP and TSI-BP’s (Possamai et al., 2024; Turnes et al., 2019). Power outputs at HHb|BP and AnT were no different between graded exercise test (GXT) types, however, wide 95% limits of agreement were reported suggesting high variance between NIRS and BLa based methods of AnT determination (Possamai et al., 2024). Overall, there was poor agreement and wide variability between MLSS/CP and NIRS-derived thresholds (Possamai et al., 2024).

Overall, the direction and rate of change of SmO_2_ during dynamic exercise tasks provide information on the balance between skeletal muscle O_2_ delivery (m $\dot{Q}$ O_2_) and local O_2_ consumption (m $\dot{V}$ O_2_) (Barstow, 2019; Kirby et al., 2021). This dynamic balance has been evaluated via comparing SmO_2_ from the work-steps of GXTs (SmO_2_rate) to BLa concentrations, with reports that changes in SmO_2_rate across a GXT may be highly related to concomitant changes in BLa (Batterson et al., 2023; Batterson et al., 2024). More recently, additional attention has been provided to the rates of SmO_2_ resaturation (SmO_2_resat) during dynamic exercise tasks, as during post-exercise recovery periods skeletal muscle’s demand for m $\dot{V}$ O_2_ is reduced. While the supply of O_2_ (m $\dot{Q}$ O_2_) also declines during rest periods, it does so at a slower rate permitting SmO_2_ to change towards a positive directionality and rise (i.e., resaturate) now that supply of O_2_ exceeds local tissue consumption (Van Beekvelt et al., 2001; Behnke et al., 2009; Arnold et al., 2024). This balance of m $\dot{Q}$ O_2_ and m $\dot{V}$ O_2_ biasing greater O_2_ delivery can be referred to as reoxygenation and/or SmO_2_ resaturation. Increases in the rate at which individuals can recover SmO_2_ during intermittent rest periods throughout repeated bouts of exercise likely reflects a combination of improved restoration of metabolic factors (e.g., phosphocreatine) (Yoshida and Watari, 1993; Pilotto et al., 2022), faster reperfusion of oxygenated blood into the microcapillary networks within skeletal muscle, and larger O_2_ delivery capacity. Thus SmO_2_rate may change as intensity scales and the demands of skeletal muscle increase throughout a GXT, especially considering the documented disproportionate alterations in the metabolic millieu within skeletal muscle at circa-maximal exercise intensities (Brooks et al., 2022; Hargreaves et al., 1998; Yoshida and Watari, 1993). Collectively, available data is conflicting on the agreement between NIRS derived breakpoints and LT_1_ and LT_2_ (Sendra-Pérez et al., 2023), with some reports of very wide limits of agreement when compared to LT_2_ specifically (Turnes et al., 2019). Additionally, less work has directly investigated changes in dynamic SmO_2_ kinetics such as SmO_2_rate and SmO_2_resat.

Therefore, the purpose of this study was to investigate the association and agreement between SmO_2_ breakpoints (SmO_2_BP1 and SmO_2_BP2) from the VL and LT_1_ and LT_2_ derived from discontinuous GXTs. In addition, longitudinal changes in the aforementioned physiological variables will be assessed across multiple time points during an entire academic year (August 2023 – May 2024). Further, secondary aims are to: 1) quantify the degree to which dynamic changes in SmO_2_ during each GXT work-step (SmO_2_rate) relate to dynamic changes in BLa kinetics, and 2) determine to what extent SmO_2_ inter-step recovery kinetics change throughout GXTs. Collectively, the forthcoming analyses aim to provide additional insight on how VL SmO_2_ kinetics change both acutely and longitudinally, as well as how SmO_2_ kinetics relate to commonly measured BLa kinetics during GXT protocols in female rowing athletes.

## Materials and methods

### Experimental approach to the problem

A combination of cross-sectional and time-series research designs were employed in the present study. Data were collected at three pre-determined times in coordination with the University of Kansas’ Women’s Rowing Coaching Staff throughout the 2023–2024 academic year (September, November, and February), in alignment with the beginning of the Fall (Sept), end of the Fall (Nov), and just prior to the beginning of the main competitive season during the Spring academic term (Feb). A subset of 11 athletes were able to complete at least two repeat NIRS-equipped GXTs, with physiological data analyzed for longitudinal changes. The remaining 12 athletes performed a single GXT at one of the three aforementioned testing times. When comparing physiological variables from a single GXT across all 23 athletes, data from each athlete’s first GXT of the 2023–2024 were used.

### Subjects

Twenty-three National Collegiate Athletic Association (NCAA) Division I female rowing athletes performed the GXT protocol outlined in the present study as part of their routine longitudinal performance monitoring. According to McKay et al.‘s proposed classification framework, these athletes are considered highly trained/national level (Tier 3) (McKay et al., 2022). All athletes were free of injury as cleared by the team’s athletic trainers. Further, all athletes were screened for alcohol and tobacco use within the past 24-h and any upper respiratory tract infection symptoms within the last 2 weeks from the date of the GXT. This study was carried out in accordance to the 1964 Declaration of Helsinki, and the protocol was approved by the University of Kansas’ institutional review board (STUDY#00150421).

### Procedures

#### GXT protocol

The discontinuous 7x4-min rowing ergometer (Concept2 RowErg, Model D, Morrisville, Vermont, United States) step test employed was developed by the Australian Institute of Sport (Rice, 2019) to individualize training zone prescription based upon BLa and power outputs across seven steps of increasing intensity and (Rice, 2019). The workloads for steps one to six are based on each individual athlete’s most recent personal best 2KErg, which was provided by members of the coaching staff from 2KErg testing conducted within the last 12 months (not their all time personal best), with step 7 being an all-out 4-min maximal effort. Throughout the 2023–2024 season, the GXT workloads at each step for each athlete were held constant, permitting changes in various physiological variables to be assessed longitudinally in response to the same protocol. The workload increases were consistent within each athlete but differed between athletes. For example, is an athlete’s 2KErg was between 6:50–7:00 their workload would begin at 130 W and increase 25 W for steps one to six, whereas an athlete with a 2KErg of 7:40–7:50 would begin at 110 W and increase 15 W for steps 1–6. The 7x4-min GXT protocol provides a completely individualized approach for each athlete. Readers are referred to the most up-to-date protocol document for a step-by-step breakdown of this test (Rice, 2019). Athletes were instructed to avoid the consumption of any caffeine 12 h prior to all tests and to consume a pre-workout snack containing 30–60g carbohydrate 30–60min before all GXTs. Further, athletes were instructed not to consume any supplements on testing days. Prior to beginning, all athletes performed a 10-min warm-up on rowing ergometers at 10–15 W below their step 1 starting wattages. Baseline BLa samples were taken before and after the 10-min warm-up.

#### Blood lactate sampling and threshold determination

Finger-stick BLa measures were performed using a single-use disposable lancet and Lactate Pro two handheld analyzer (Arkray, Kyoto Japan), which has been shown to have strong intra-device reliability with intraclass correlation coefficients (ICC) = 0.99, with a coefficient of variation (CV) = 3.3% and 95% limits of agreement (LoA) between −1.0 and 1.0 mmol L^-1^, making it ideal for testing in large team scenarios (Crotty et al., 2021). Prior to the beginning of the GXT, all athletes performed a 10-min warm-up on rowing ergometers at 10–15 W below their step 1 starting wattages. Baseline BLa samples were taken before and after the 10-min warm-up. All sampling was performed during the 1-min rest intervals of the discontinuous 7x4-min GXTs, with an additional sample taken 4 min after the maximal step to identify changes in BLa concentrations; the higher of the two readings (post-seventh step and +4 min) was used for each partcipant’s peak BLa concentration and later used for determination of LT_1_ and LT_2_. Finger-stick samples were taken following cleansing of the site with an alcohol pad, with the first blood droplet wiped away (KimWipe, Kimberly-Clark Professional, Roswell GA) and the BLa test strip then touched to the second blood droplet. BLa thresholds were calculated using ADAPT software (Sport Alo, 1995) and were defined as follows (Bourdon, 2013): Lactate Threshold 1 (LT_1_) is the intensity preceding a 0.4 mmol⋅L^-1^ increase of BLa above baseline values and Lactate Threshold 2 (LT_2_) is identified using the Modified DMax method, which draws a line from LT_1_ to the finishing intensity and is the maximal distance of the line from the BLa curve (Bishop et al., 1998; Bourdon, 2013; Cheng et al., 1992).

#### NIRS data collection and preparation

Portable NIRS devices (MOXY Monitor, Fortiori Design LLC, Minnesota United States) were set to sample at a rate of 1 Hz (MOXY’s “medium” setting) and affixed on the right VL at 1/3 the distance between the anterior superior iliac crest and the proximal, lateral-most patellar tip with CoverRoll stretch tape (Feldmann et al., 2019). The MOXY device uses an emitter with wavelengths at 690, 720, 760, and 800nm along with detectors placed 12.5 and 25mm from the emitter (McManus et al., 2018) providing real time data on the dynamic changes in skeletal muscle oxygenation saturation (SmO_2_). SmO_2_ is defined as the percentage of hemoglobin and myoglobin that is actively carrying oxygen within the capillaries and tissue of the muscle under investigation and is represented on a 0%–100% scale (Fortiori Design., 2016). NIRS data from all GXT trials were downloaded as Microsoft Excel files from the VO2 Master cloud software, and rowing ergometer power data was obtained from the rowing athlete management software system Ludum (Cambridge, UK).

#### NIRS inter-step recovery and work-rate parameters

**FIGURE 1:**
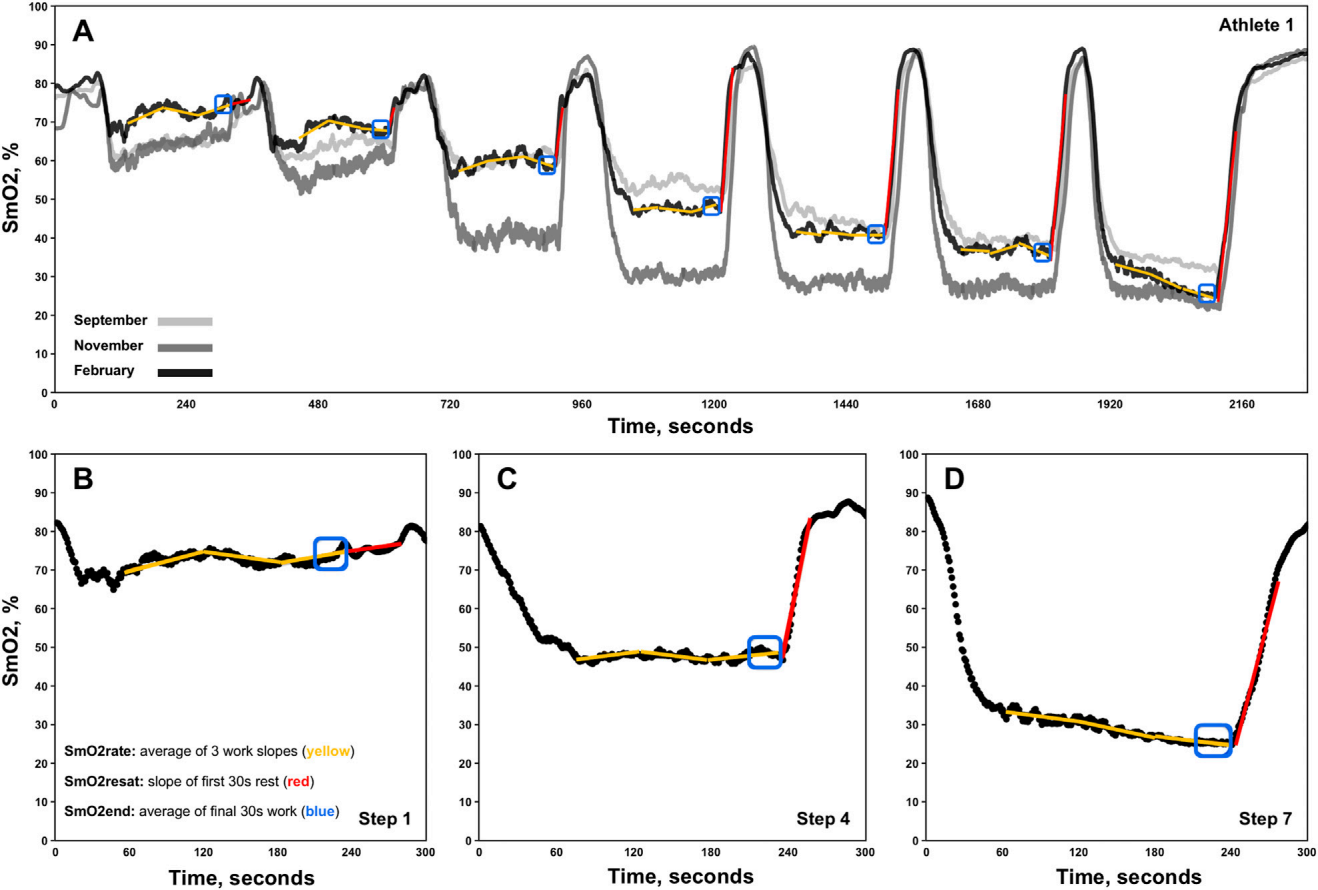
Representative single athlete data showing skeletal muscle oxygen saturation (SmO_2_) trends for the right vastus lateralis across three discontinuous 7x4-minute graded exercise tests **(A)**. Zoomed-in views of data from steps 1, 4, and 7 for the same athlete are also displayed **(B–D)**. The three linear regression analyses performed for the determination of each work-step’s SmO_2_rate are shown via segmented yellow slope lines. Red slope lines indicate SmO_2_ re-saturation rate (SmO_2_resat) slope calculations. SmO_2_end locations are also denoted in blue boxes.

SmO_2_ resaturation rates (SmO_2_resat) were calculated as the positive slope of the first 30s of SmO_2_ data during each 1-min inter-step rest interval (Figure 1. Red lines) (Eserhaut et al., 2024). Exploratorily, mean SmO_2_resat was calculated as a representative measure of local skeletal muscle *recovery capacity* across the entire GXT for each athlete with at minimum two sets of GXT NIRS data (n = 11). For SmO_2_rate, the first 60s of each work step was ignored to avoid the undue influence that the SmO_2_ onset kinetics would have on subsequent calculations (Batterson et al., 2023). Three slopes were calculated for the remaining 60-s segments of each work step (e.g., 61–120, 121–180, 181–240s), with SmO_2_rate for each step representing the mean of these three work slopes (Figure 1. Yellow segmented lines). To determine both the degree of association and the shape of the relationships between SmO_2_rate and BLa, as well as SmO_2_rate and power, linear regression, second order polynomial, and inverse regression models were fit to the mean values for SmO_2_rate and BLa across all 7-steps for 23 sets of GXT data obtained, aligning with the first NIRS-equipped GXT performed by each athlete. Bayesian information criterion (BIC) and coefficients of determination (R^2^) determine the best model fits (Chakrabarti and Ghosh, 2011). The mean SmO_2_ from the final 30s of each work step (e.g., 210–240s) was calculated (SmO_2_end) for use in the forthcoming breakpoint analyses (Figure 1. Blue boxes).

#### NIRS breakpoint estimation

To determine SmO_2_ breakpoints (SmO_2_BP1 and SmO_2_BP2) piecewise linear regression models were fit to the variables SmO_2_end and mean power (W), from the first GXT of the season performed by each individual athlete using the “segmented” package (version 2.1.0). Each piecewise model was specified to contain two breakpoints (npsi = 2) prior to estimation. Briefly, the “segmented” package fits each model by minimizing the sum of squared residuals and testing for changes in linear slopes, providing estimated locations at which the slope of the relationship between y-axis variable (SmO_2_end) and x-axis variable (power) change to a statistically significant degree. Further details on the piecewise linear regression method used can be found in Muggeo, 2003) with a description of the “segmented” R-package (v2.1.0) available in Muggeo, 2008). Once breakpoint estimations were returned, researchers visually verified the breakpoint estimations via creating a scatter plot of SmO_2_end x Power overlayed with the segmented linear regression model.

### Statistical analyses

All statistical analyses and data visualization were performed in R Studio (version 1.4.1106). A 1 × 7 repeated measures analysis of variance (RMANOVA) was used to determine if SmO_2_resat exhibited intensity dependent changes across the 7x4-min GXT protocol. Linear mixed-effects models were fit using the “lme4” package (version 1.1.34) to investigate mean differences (MD) in the variables mean SmO_2_resat, LT_1_, LT_2_, SmO_2_BP1, and SmO_2_BP2 across the fixed factor of time (e.g., September, November, February), using the individual athlete as a random factor [e.g., meanSmO_2_resat ∼ Time + (1 | ID)]. Within these models, restricted maximum likelihood (REML) estimates were used to account for missing repeated measures (Enders, 2006), with standard errors of the estimates (SEE) provided for the estimated mearginal means of mean SmO_2_resat, LT_1_, LT_2_, SmO_2_BP1, and SmO_2_BP2. Following significant univariate effects for time, *post hoc* Tukey HSD tests were performed.

Paired-samples t-tests were used to test for significant MD in LT_1_ and SmO_2_BP1, along with LT_2_ and SmO_2_BP2, with accompanying Cohen’s *d* effect sizes. Effect size interpretation thresholds of small (0.2), medium (0.5), and large (0.8) were used (Cohen, 1988). Further, the two one-sided tests (TOST) procedure was used to test for statistical equivalence between LT_1_ and SmO_2_BP1, and LT_2_ and SmO_2_BP2 via the “TOSTER” package (version 0.8.3) (Lakens et al., 2018). Upper and lower equivalence bounds (∆_U_ and ∆_L_) were set to the *a priori* critical effect size for our study of *d* = 0.6435, given n = 21, our critical t-statistic of 2.086, and power (1-β err prob) of 0.8 (Lakens et al., 2018). *Bland*-Altman plots comparing LT_1_ and SmO_2_BP1, and LT_2_ and SmO_2_BP2 were created using the “BlandAltmanLeh” package (version 0.3.1), with linear regressions fit to the means and differences for LT_1_ and SmO_2_BP1, and LT_2_ and SmO_2_BP2, describing potential relationships between the direction of measurement biases and the magnitude of the thresholds. In addition to the Bland-Altman analyses, intraclass correlation coefficients were calculated using a single-measure, two-way mixed-effects model (ICC_2,1_) to determine absolute agreement between the two physiological measurement methods using the “psych” package (version 2.4.6.26), with 95% confidence intervals (95% CI) also calculated (Shrout and Fleiss, 1979; Weir, 2005). Interpretations of poor (<0.5), moderate (0.5–0.75), good (0.75–0.9)m and excellent (>0.9) were used (Koo and Li, 2016). Pearson product-moment correlations were also performed between LT_1_ and SmO_2_BP1, and LT_2_ and SmO_2_BP2 to determine the degree of association between the variables. Statistical interferences for all analyses were made using an *a priori* alpha level of p ≤ 0.05.

## Results

This cohort of NCAA Division I female rowers had 2KErg times ranging from 6:58.8 to 7:58.4 and had 3.0 ± 2.2 years of on-water and indoor rowing experience. Further descriptive statistics are reported in Table 1.

**TABLE 1:** Participant characteristics.

Variable	Mean ± SD
N	23
Age (years)^a^	20.0 (18–22)
Height (cm)	176.3 ± 4.4
Body Mass (kg)	74.4 ± 9.2
On-Water Rowing Experience (years)	3.0 ± 2.2
Indoor Rowing Experience (years)	3.0 ± 2.2
2,000-m Watts (W)^a^	227 (205–305)

^a^ Non-parametric variables, which are reported as median (range).

### Acute SmO_2_ resaturation rates

For SmO_2_resat from the first NIRS equipped-GXT of the season (*n* = 23), a significant main effect for Time was present (η^2^_p_ = 0.71, p < 0.001), with SmO_2_resat clearly displaying marked intensity-dependent increases, as Tukey HSD *post hoc* comparisons between all steps yielded significant differences in SmO_2_resat (p < 0.01), with the exception of step 5 – step 6 (p = 0.064), step 5 – step 7 (p = 1.000), and step 6 – step 7 (p = 0.609).

### Longitudinal changes in mean SmO_2_ resaturation rates

**FIGURE 2:**
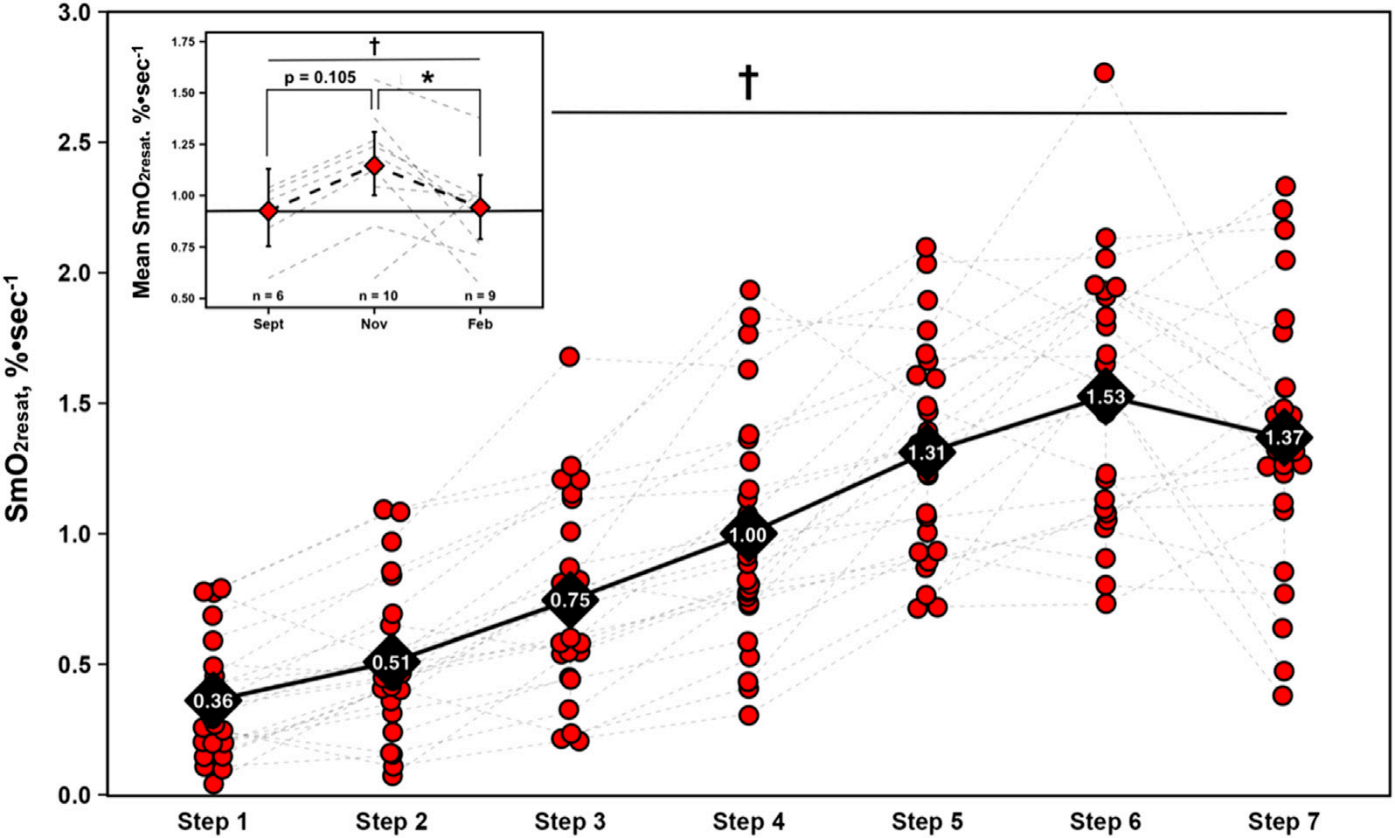
Skeletal muscle oxygen re-saturation rates (SmO_2_resat) calculated as the slope of the first 30s of SmO_2_ data during each 1-min inter-step rest interval from the first GXT completed with NIRS for n = 23 athletes. Individual data shown via red dots and gray dashed spaghetti plots. Black diamonds display mean values for each work-step, connected via solid black line. Top left figure inset presents longitudinal changes in mean SmO_2_resat for a subset of athletes (total n = 11). Individual spaghetti plots are gray dashed lines, with estimated marginal means shown as red diamonds ±95% confidence intervals from the linear mixed-effects model, connected via black dashed line. Solid black line indicate Sept’s means, treated as the baseline reference. † = main effect for Time (p ≤ 0.05); * = significant difference (p ≤ 0.05).

A significant univariate effect for mean SmO_2_resat was present (F = 4.35, p = 0.033), indicative of changes across time. Tukey HSD *post hoc* comparisons yielded a significant reduction from Nov to Feb (MD = −0.21, SEE = 0.08, df = 13.5, t = 2.69, p = 0.044). Of note is the nearly significant elevation in mean SmO_2_resat from Sept-Nov (MD = 0.21, SEE = 0.10, df = 15.0, t = 2.19, p = 0.105) that preceded this significant decline. Changes in both SmO_2_resat during a single GXT and mean SmO_2_resat across the season are shown in Figure 2.

### SmO_2_ work rates

**FIGURE 3:**
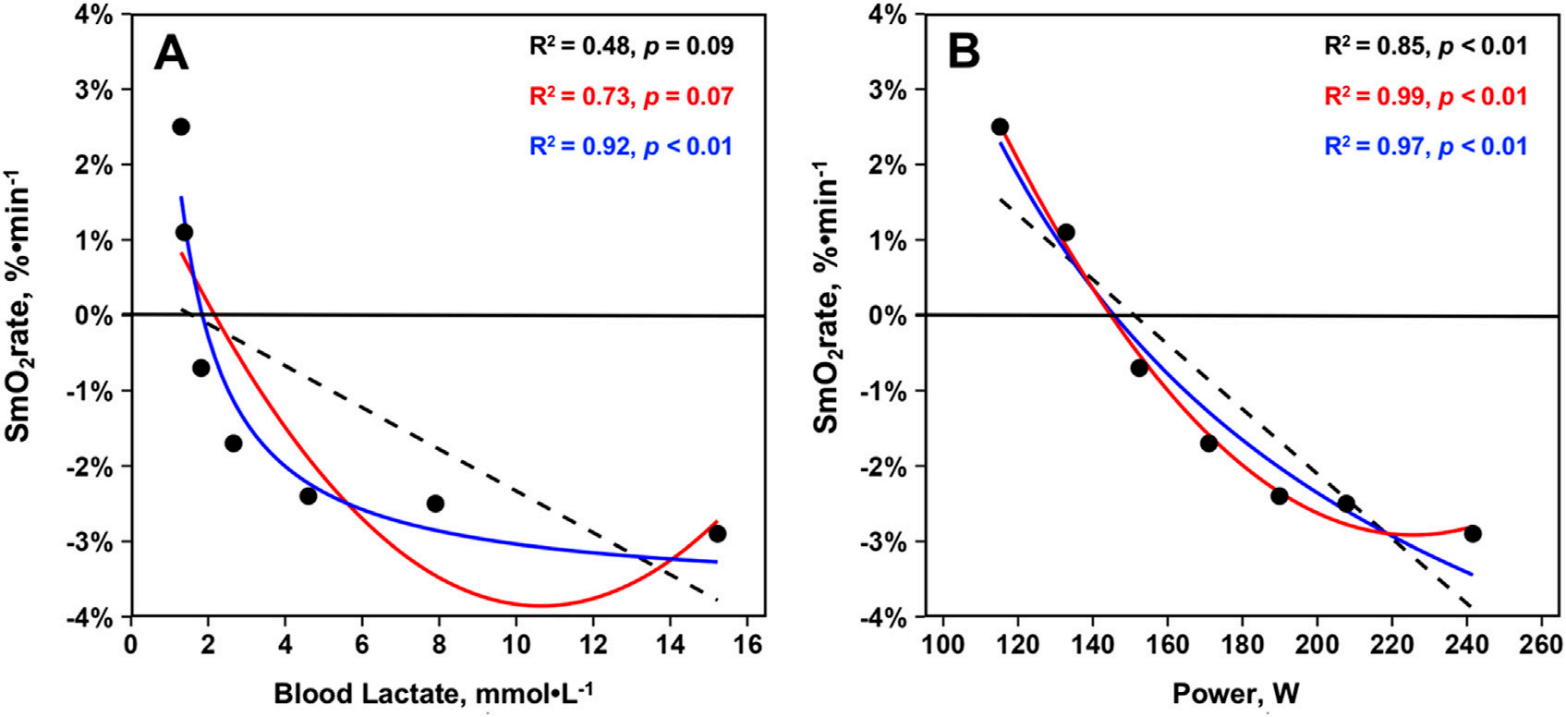
Mean skeletal muscle oxygen saturation rates (SmO_2_rate) from the right vastus lateralis during the seven work-steps plotted against **(A)** mean blood lactates and **(B)** mean power outputs from the first GXT completed with NIRS for n = 23 athletes during the 2023–2024 season (23 total trials). Model fits displayed are simple linear regressions (black, dashed), second order polynomials (red, solid), and inverse regressions [formula: y ∼ I (1/x)] (blue, solid). Variance explained by each model fit with statistical significance is displayed.

**FIGURE 4:**
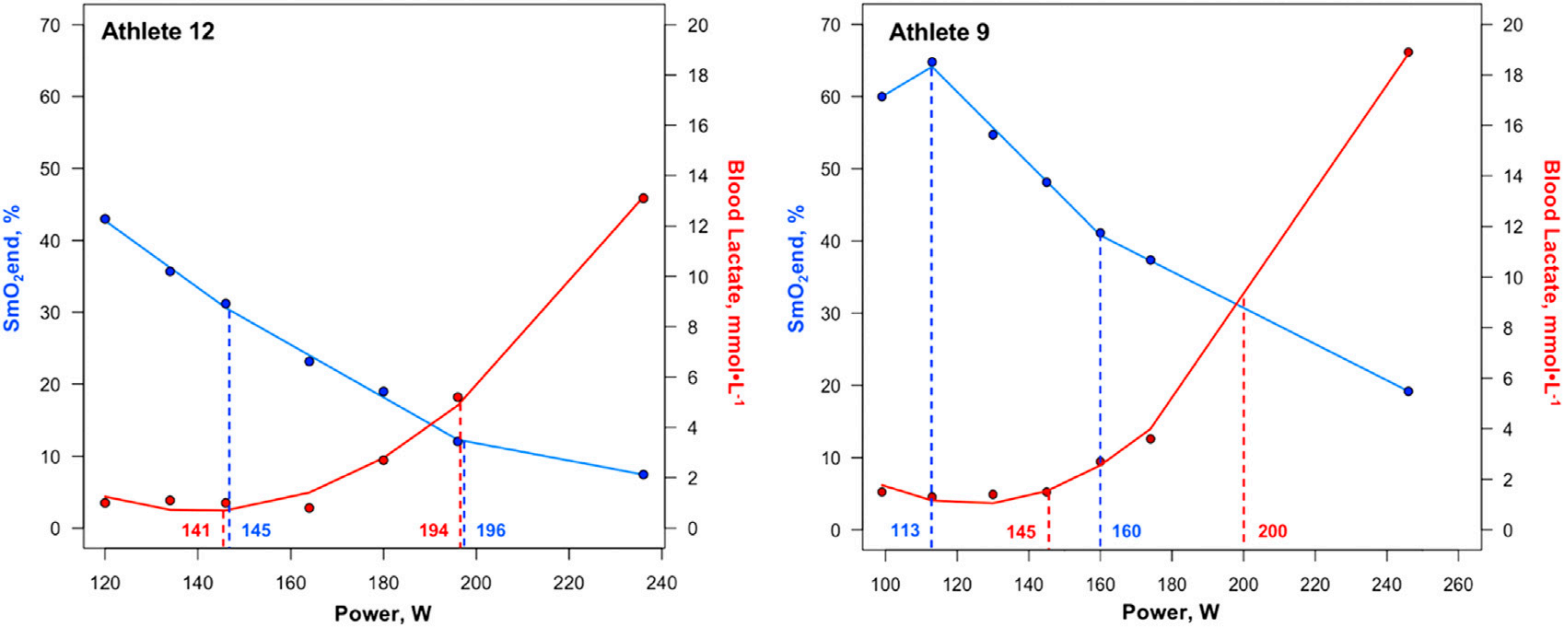
First and second SmO_2_ breakpoints and lactate thresholds shown for athletes with the greatest agreement (Athlete 12) and least agreement (Athlete 9) between the two methods of physiological threshold determination. Solid blue lines represent segmented regression analyses, with dashed blue lines and accompanying power values demarcating SmO_2_BP1 and SmO_2_BP2. Solid red lines represent lactate curves generated using the ADAPT software, with dashed red lines and accompanying power values demarcating LT_1_ and LT_2_. SmO_2_BP = skeletal muscle oxygen saturation breakpoint; LT = lactate threshold.

Relationships between mean SmO_2_rate, BLa, and power across the GXT protocol from 23 athletes are displayed in Figure 4. For SmO_2_rate’s relationship with BLa, linear regression (R^2^ = 0.48, p = 0.09, σ = 1.62, BIC = 30.10) and second order polynomial (R^2^ = 0.73, p = 0.07, σ = 1.30, BIC = 27.40) model fits were not statistically significant. However, plotting SmO_2_rate against the inverse of BLa led to a strong statistically significant curvilinear fit (R^2^ = 0.92, p < 0.01, σ = 0.64, BIC = 17.10). For the relationship between SmO_2_rate and power, all models were significant. BIC and R-squared values indicated that a curvilinear second order polynomial best explains the relationship between SmO_2_rate and power. Linear regression (R^2^ = 0.85, p < 0.01, σ = 0.86, BIC = 21.20), second order polynomial (R^2^ = 0.99, p < 0.01, σ = 0.20, BIC = 1.31), and inverse regression (R^2^ = 0.97, p < 0.01, σ = 0.41, BIC = 10.80) all explained a significant proportion of the variance. The model fits are shown in Figure 3.

### Differences and agreement between SmO_2_ breakpoints and lactate thresholds

A significant difference was present between LT_1_ and SmO_2_BP1 (t = −2.41, MD = −8.14 [95% CI: −15.2 to −1.1], *d =* 0.525, p = 0.026), with poor-to-moderate agreement between the two measurement methods (ICC_2,1_ = 0.24 [95% CI: −0.13–0.58], p = 0.10). TOST equivalence testing also found LT_1_ and SmO_2_BP1 were not statistically equivalent (t_(40)_ = 0.032, p = 0.487, ∆_U_ and ∆_L_ = −8.33–8.33 W). Conversely, there was no significant difference between the higher intensity thresholds LT_2_ and SmO_2_B2 (t = −1.56, MD = −5.76 [95% CI: −13.5 to 1.93], *d =* 0.341, p = 0.134), with these higher intensity thresholds possessing moderate-to-good levels of agreement (ICC_2,1_ = 0.67 [95% CI: 0.36–0.85], p < 0.001). However, TOST equivalence testing found LT_2_ and SmO_2_BP2 were not statistically equivalent (t_(40)_ = 1.207, p = 0.117, ∆_U_ and ∆_L_ = −13.77–13.77 W). Figure 4 shows the SmO_2_BP1 and SmO_2_BP2 and LT_1_ and LT_2_ for the athletes with the greatest agreement and least agreement between the two methods of physiological threshold determination. Additionally, individual lactate threshold and SmO_2_ breakpoint data for all athletes is displayed in Table 2.

**TABLE 2:** Individual SmO_2_ breakpoints compared to lactate thresholds.

Athlete	Power output, W		Power output, W	
	SmO_2_BP1	LT1	SmO_2_BP2	LT2
1	144	160	190	212
2	144	176	236	244
3	141	131	216	202
4	148	176	195	234
5	148	157	188	208
6	115	126	145	158
7	134	144	165	183
8	159	148	191	178
9	113	145	160	200
10	156	141	185	179
11	128	149	204	198
12	145	141	196	194
13	-	-	-	-
14	131	137	164	167
15	152	142	179	188
16	140	135	192	183
17	133	141	181	182
18	143	144	182	174
19	133	129	167	169
20	-	-	-	-
21	131	168	169	197
22	148	150	215	210
23	134	151	187	168
Mean ± SD	139.0 ± 11.9*	147.2 ± 13.9	186.0 ± 21.0	191.8 ± 21.8

Individual threshold data presented are from the first test performed with NIRS by each athlete during the 2023–2024 season. “-” denotes NIRS data inadequate for piecewise linear regression analysis with two predetermined breakpoints. SmO_2_BP = skeletal muscle oxygen saturation breakpoint; LT = lactate threshold; * = significant difference from LTs (p ≤ 0.05).

**FIGURE 5:**
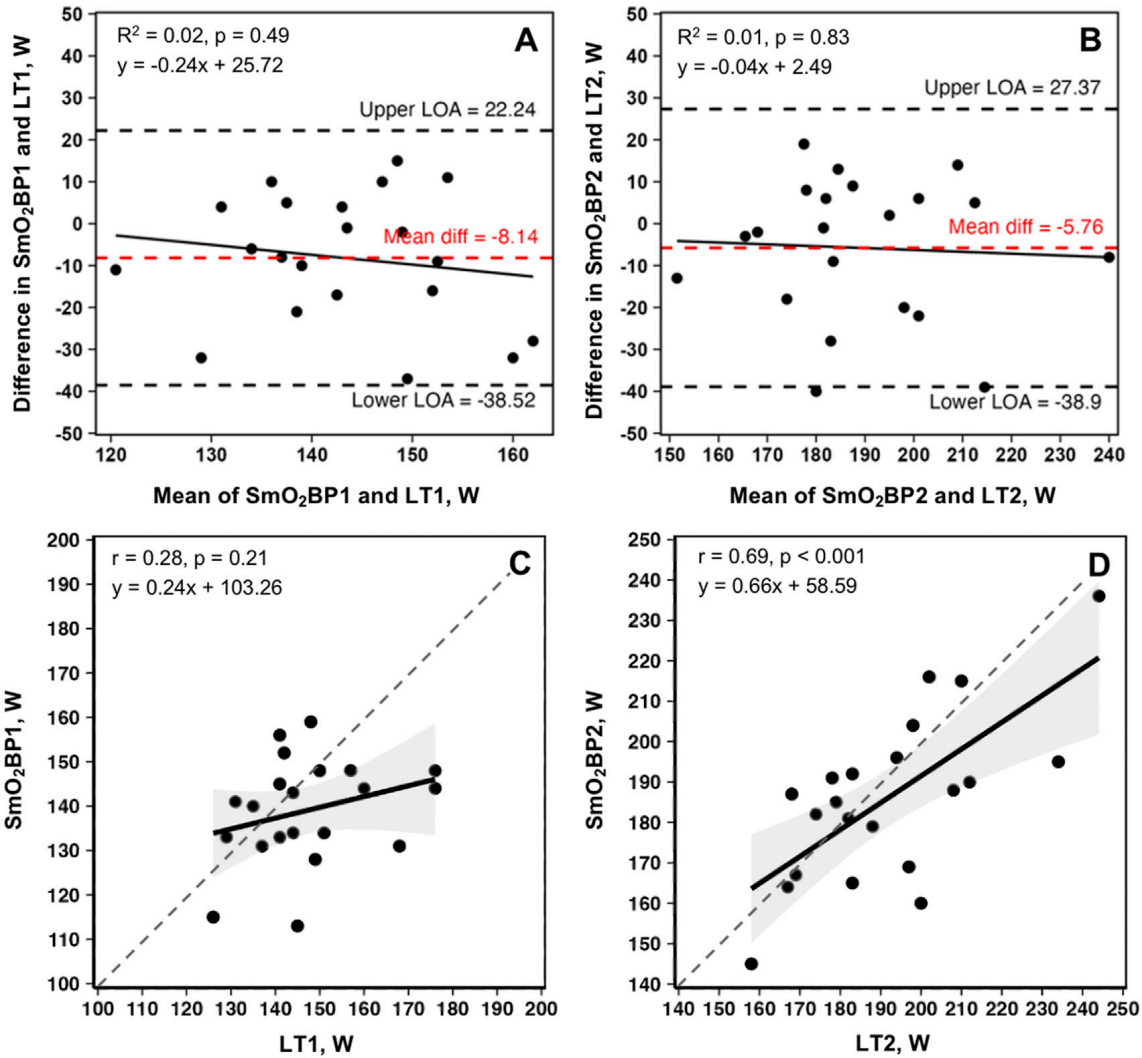
Bland-Altman plots and linear regressions for SmO_2_BP1 and LT_1_ **(A, C)**, and SmO_2_BP2 and LT_2_ **(B, D)** from the first NIRS equipped GXTs of the season for n = 21 athletes (21 total trials: one per athlete). Dashed red lines represent mean biases, with dashed black lines representing upper and lower 95% limits of agreement (LoA). Solid black lines show linear regressions, with gray dashed lines of agreement. Gray shaded areas are 95% confidence intervals. SmO_2_BP = skeletal muscle oxygen saturation breakpoint; LT1 = lactate threshold.

Bland-Altman plots displaying mean biases and 95% limits of agreement (95% LoA), along with the relationships between the two methods of physiological threshold determination, are displayed in Figure 5.

### Longitudinal changes in SmO_2_ breakpoints and lactate thresholds

**FIGURE 6:**
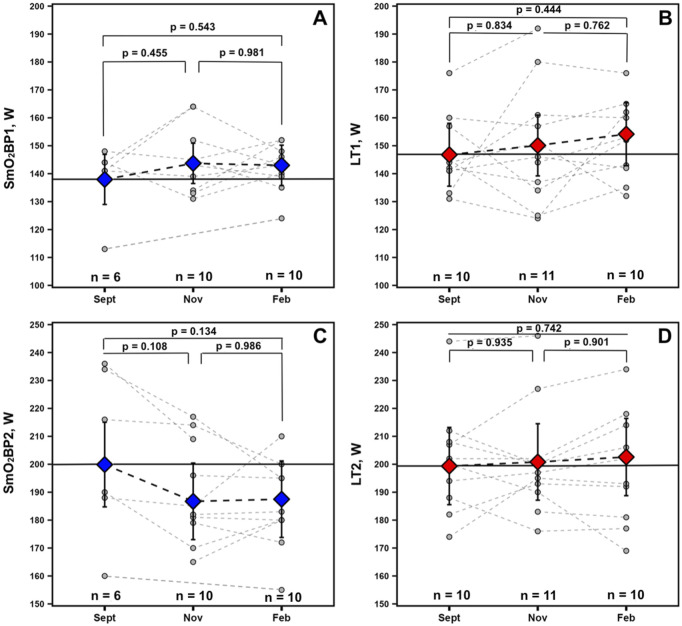
Data for athletes with at minimum 2 sets of GXT NIRS data are displayed. Individual spaghetti plots are gray dashed lines, with estimated marginal means ±95% confidence intervals from the linear mixed-effects models for SmO2 breakpoints 1 and 2 (blue: **A, C**) and BLa thresholds 1 and 2 (red: **B, D**), connected via black dashed lines. Solid black lines indicate Sept’s estimated marginal means, treated as the baseline reference.

For the physiological thresholds determined, no significant univariate effects were present for any of the measured variables indicative of no statistically significant changes across the season. However, a trend toward a univariate effect for Time was present for SmO_2_BP2 (F = 2.715, p = 0.102). Estimated marginal means and 95% confidence intervals from the mixed-effects models along with p-values from all non-significant Tukey HSD-adjusted pairwise comparisons are displayed in Figure 6 for descriptive purposes.

## Discussion

The purpose of this study was to investigate longitudinal changes in NIRS breakpoints and SmO_2_ trends during GXT’s performed across multiple time points during an entire academic year (August 2023– May 2024), along with determining the degree of agreement between NIRS and BLa methods of physiological intensity threshold identification. To the best of our knowledge, our study is the first to include longitudinal NIRS data on female rowers. The majority of NIRS rowing studies have focused on one to two trials to identify either HHb or TSI/SmO_2_ breakpoints with most participants in these studies being male rowers (Possamai et al., 2024; Lanigan, 2022; Turnes et al., 2019; Klusiewicz et al., 2021). Further, SmO2resat kinetics were also investigated for intensity-dependent changes and a possible high-intensity induced blunting effect. Exploratory, SmO2resat values were averaged across GXTs for the athletes with longitudinal NIRS data and evaluated as a more generalized indicator of local muscle recovery capacity. Lastly, the dynamic intra-exercise balance between mQ̇O_2_ and mV̇O_2_ was (SmO_2_rate) was compared to mean BLa concentrations across this cohort of female rowers to provide insight into the shape of the relationship between SmO_2_ kinetics during GXT work-steps and BLa concentrations per prior reports of linear and subtle curvilinear relationships in elite male soccer athletes (Batterson et al., 2023; Batterson et al., 2024).

The principal findings with the greatest applied significance are that no difference was found between SmO_2_BP2 and LT_2_ (mean diff = −5.76W [95% LoA = −38.52 to 22.25W], p = 0.134), with moderate-to-good levels of agreement between the two (ICC_2,1_ = 0.67 [95% CI: 0.36–0.85], p < 0.001), and a significant positive relationship (r = 0.69, p < 0.001).

This was not the case for SmO_2_BP1 and LT_1_, with NIRS significantly underestimating LT_1_ (mean diff = −8.14W [95% LoA = −38.90 to 27.37W], p = 0.026), with poor-to-moderate agreement (ICC_2,1_ = 0.24 [95% CI: −0.13–0.58], p = 0.10). Taken together, NIRS derived SmO_2_ breakpoint determination does not appear to be a viable alternative for LT_1_ determination, and despite the lack of a statistically significant difference in SmO_2_BP2 and LT_2_, notable inter-individual variance in agreement raises concerns about the use of NIRS as a standalone tool for training zone intensity prescriptions centered around LT_2_. Figure 4 highlights the range of differences between LT and SmO_2_ breakpoints at the individual level, as from an applied perspective, if athlete 9 (panel B) utilized the SmO_2_BP2 to anchor training session intensity, this would result in a markedly different training stimulus than their LT_2,_ resultantly different adaptations. Further, the piecewise linear regression modeling method used could not fit two sets of SmO_2_ data from the 23 GXT trials analyzed for physiological breakpoints. This suggests an 8.7% failure rate with the NIRS methods employed in this investigation when specifying two breakpoints are present prior to modeling.

The trend of NIRS underestimating the lactate thresholds measured could be in part due to limited oxygen availability within skeletal muscle preceding subsequent BLa accumulation. Thus, an inability to supply O_2_ in quantities sufficient enough to match increasing O_2_ demands locally within the skeletal muscle tissue (i.e., m $\dot{Q}$ O_2_) during higher intensitiy exercise limits the availability of O_2_ within the vastus lateralis microcapillaries, evidenced by low absolute SmO_2_ values (see step 7 in Figures 1, 4), which may serve as a rate-limiter in the mitochondrial oxidation of BLa and contribute to exponential rises in circulating BLa concentrations at higher exercise intensities. Thus, with limited O_2_ availability within skeletal muscle microcapillaries may provide some physiological rationale as to why SmO_2_BP2 trended towards underestimating LT_2_. As for the significant underestimating of LT_1_ by SmO_2_BP1, rather than a physiological reason, we posit that athletes expressing a positive SmO_2_ curve during early GXT stages of the GXT where m $\dot{V}$ O_2_ is greater than m $\dot{Q}$ O_2_ during these lower exercise intensities biased some of the SmO_2_BP1 estimations towards the left (see Figure 4, panel B, SmO_2_BP1). Such positive SmO_2_ curves have previously been reported during prolonged discontinuous bouts of mixed-intensity cycling where an increase in the absolute SmO_2_ values occurs as exercise progresses (Yogeve et al., 2023) indicating that a given individual is capable of supplying greater quantities of O_2_ than is being demanded by the exercising musculature yielding increased available O_2_ within muscle microcapillaries during exercise. This increase in SmO_2_ has early on also been observed during treadmill GXTs where SmO_2_BP1 was estimated to be in close proximity to this event in some athletes (Batterson et al., 2023). Thus, estimation of LT_1_ using SmO_2_ may be prone to inter-individual variances in their ability to deliver O_2_ to exercising musculature during lower exercise intensities. While our chosen 7x4-min GXT offers individualized starting intensities for each athlete (Rice, 2019), it may still be challenging to align step 1 with a wattage that yields a neutral to negative SmO_2_rate throughout this 4-min piece with a high degree of consistency allowing practioners to avoid this positive SmO_2_ curve at the start of testing. Further, when testing for statistical equivalence a very forgiving range of equivalence bounds (∆_U_ and ∆_L_ = −13.77–13.77 W) were used based upon our *a priori* critical effect size (*d =* 0.6435) calculated following methods of Lakens et al. (2018). Results showed a lack of equivalence between SmO_2_BP1 and LT_1_, and, despite a null mean difference between SmO_2_BP2 and LT_2_, no statistical equivalence was found for these higher intensity physiological threshold measures either, providing evidence that NIRS breakpoints, which are derived from local O_2_ supply and demand kinetics within the skeletal muscle tissue of interest, are not inter-changeable with systemic BLa thresholds. This is not to say that SmO_2_BP’s and LT’s, which both quantify notable metabolic events during exercise, are not related. Indeed, these significant inflections in metabolic markers likely occur in a series of cascading events that take place during transitions across the exercise intensity spectrum and may influence one-another to some degree (Boone et al., 2016). However, despite SmO_2_ and BLa kinetics being associated with one another physiologically, the two methods of monitoring human metabolism during exercise do not appear to be one and the same.

Of additional importance with respect to the logistics regarding data collection is that blood samples were taken during the 1-min rest period and are representative of BLa that has diffused into the capillaries at that moment in time compared to the NIRS breakpoints, which are the mean SmO_2_ from the final 30s of each work step measured in real-time. The delay in time to collect the blood sample may be related to the differences between BLa and NIRS breakpoints as BLa levels have been shown to be dependent on the time to capture post-exercise, with longer post-exercise measurement times leading to lower BLa values (Vucetic et al., 2015). Furthermore, BLa threshold determinations are highly dependent on the structure of the GXT protocol used and the method chosen to calculate LT_1_ and LT_2_ (Jamnick et al., 2018). Importantly, NIRS thresholds likely also possess some degree of dependancy on the structure and design of the GXT protocol chosen, and the breakpoint estimation method employed. For example, continuous GXTs yield an unbroken time-series of SmO_2_ data (Feldmann et al., 2022) providing a greater quantity of data points to fit piecewise linear regression models which may produce SmO_2_BP values that differ to some degree from those derived from discontinuous GXTs often required to conduct BLa testing during rowing ergometer trials.

The second finding of this investigation was that our cohort of NCAA Division I female rowers displayed a very curvilinear relationship between VL SmO_2_rate and BLa. While this finding appears to agree with data from Batterson and colleagues, the shape of our regression model fits for the VL are dissimilar to the marked linear and subtle curvilinear relationships between SmO_2_rate and BLa concentrations found during treadmill GXTs in elite male soccer athletes (Batterson et al., 2023; Batterson et al., 2024). This cohort of female rowers showed marked declines in SmO_2_rate during the early steps of the discontinuous 7x4-min GXT despite minimal changes in BLa, with the best model fit being very much curvilinear in nature (inverse regression: R^2^ = 0.92, p < 0.01). This indicates SmO_2_rate does not, on average, become increasingly negative above BLa levels of approximately 4–5 mmol⋅L^-1^ (i.e., GXT Step #5), with SmO_2_rate plateauing at a fairly stable −2.0%-3.0%⋅min^-1^ throughout the remainder of the GXT in this cohort of female athletes (see Figure 3). Such a curvilinear relationship between SmO_2_rate and BLa accumulation is akin to data from the long-head of the BF in other reports (Snyder and Parmenter, 2009; Brooks et al., 2022), a muscle that may reside under greater thicknesses of adipose tissue compared to the VL (Ishida et al., 1995). Further, sex differences in NIRS-derived values have been reported during vascular occlusion tests, with men desaturating and resaturating the VL at faster rates than women (Keller et al., 2023). Thus, the shape of the relationship between SmO_2_rate and BLa accumulation throughout GXTs may differ as a function of sex, training status, exercise modality, GXT structure, or variance in adipose tissue thickness.

The final finding of this study was that SmO_2_resat expresses intensity-dependent changes during graded exercise testing. With SmO_2_ defined as the percentage of hemoglobin and myoglobin that is carrying oxygen to the capillaries (Fortiori Design, 2016), SmO_2_resat represents the rate at which oxygen is replenished to the local muscle tissue (VL) during the 1-min rest intervals relative to the local skeletal muscle tissue’s uptake of O_2_. After the all-out maximal efforts in step 7, SmO_2_resat was no different from the sub-maximal workloads in step 6 (p = 0.694), demarcating a change in the statistically significant rate increases in SmO_2_resat from steps 1–5. These findings are in agreement with recent findings by Arnold et al., 2024; Arnold et al., 2024). We posit this is potentially due to marked elevations in BLa, hydrogen ions, and overall disruption to the metabolic millieu within skeletal muscle at higher exercise intensities (Brooks et al., 2022; Hargreaves et al., 1998; Yoshida and Watari, 1993). High-intensity exercise also induces elevations in glycolysis leading to marked increases in acetyl-coenzyme A (acetyl-CoA), and, thereby, the formation of greater amounts of malonyl-coenzyme A (malonyl-CoA) (Brooks et al., 1999). Malonyl-CoA has been shown to downregulate the rate-limiting enzyme for mitochondrial β-oxidation of free-fatty acid derivatives, and as a result, oxygen is likely left available for use in transferring BLa into the mitochondrial matrix (Mcgarry et al., 1977; Saddik et al., 1993). We speculate that the blunting in SmO_2_resat observed following the GXTs maximal seventh step effort may, in part, be related to the oxidative shuttling of BLa into the mitochondrial matrix for use as a readily available and abundant energy substrate during the immediate post-exercise recovery period. These same physiological factors may also explain, to some degree, the nonsignificant trend towards SmO_2_BP2 underestimating LT_2_.

Furthermore, when averaged across the GXT and monitored longitudinally, mean SmO_2_resat may be a variable worth monitoring for positive and negative adaptations to training programs and could serve as a sensitive proxy for short-to-moderate term fluctuations in training load. Athletes had nearly significant SmO_2_resat elevations in Nov (p = 0.105) when their volumes of training were the highest with their low-intensity sessions completed below LT_1_; these returned to near baseline values following winter break in Feb after a high-intensity training block with reduced volume. For the physiological thresholds LT_1_ and SmO_2_BP1, along with LT_2_, gradual increases were observed throughout the season. However, the magnitudes of these improvements were not statistically significant in this cohort. Interestingly, SmO_2_BP2 appears to have declined throughout the season, the inverse of LT_2_. For mean SmO_2_resat specifically, this may potentially reflect acute adaptation to the training program followed by some degree of detraining from winter break or a response to the high-intensity training block over the winter (see Figure 2). Increases in the rate at which individuals can recover SmO_2_ during intermittent rest periods throughout repeated bouts of exercise likely reflects a combination of improved restoration of metabolic factors (e.g., phosphocreatine) (Yoshida and Watari, 1993; Pilotto et al., 2022), faster reperfusion of oxygenated blood into the microcapillary networks within skeletal muscle, and larger O_2_ delivery capacity (Barstow, 2019). While mean SmO_2_resat is an exploratory metric calculated by averaging the derived SmO_2_resat metric from all seven post-step recovery windows, it may still provide some degree of signal into changes in the aforementioned physiological characteristics.

### Limitations and future recommendations

First, skinfold thickness and/or adipose tissue measurements at the site of the NIRS device were not obtained. SmO_2_ variables can be significantly skewed in individuals with skinfold thicknesses above 15mm (Feldmann et al., 2019). Thus, follow-up investigations should aim to monitor the variables presented in this investigation with a homogenous cohort of athletes with known skinfold thicknesses of sub-15mm, as inter-individual variances in skinfold thickness may introduce undesired variance into the data. Second, not all 23 athletes were able to perform two or more GXTs while equipped with a NIRS device due to various logistical challenges. Thus, future longitudinal studies should target larger sample sizes to provide increased clarity on the alterations in the investigated variables over time. Finally, while energy intake and exercise energy expenditure were not accounted for, the prevalence of relative energy deficiency in endurance sport athletes (REDs) (Mountjoy et al., 2023; Dasa et al., 2024), especially in females, may also impact SmO_2_ kinetics. Finally, measures of SmO_2_ recovery kinetics during discontinuous exercise (e.g., SmO_2_resat and mean SmO_2_resat) should be investigated in future studies to better elucidate their responsiveness to different training interventions, and potential utility as indicators of local physiological adaptations.

## Conclusion

To the best of our knowledge, this is the first study monitoring SmO_2_ breakpoints longitudinally across multiple GXTs in a cohort of highly trained female athletes. Wearable NIRS technology appears to be a non-invasive complimentary tool to BLa testing when worn during the chosen discontinuous 7x4-min GXT protocol, which is in agreement with prior research (Turnes et al., 2019). This is not the case for the lower intensity LT_1_, as NIRS significantly underestimated LT_1_ in this population. Importantly, equivalence testing revealed that SmO_2_ breakpoints and BLa thresolds are not statistically equivalent, providing evidence that the two are not interchangeable. Further analysis of SmO_2_ kinetics show changes in SmO_2_rate display clear curvilinear relationships with BLa concentrations throughout the rowing ergometer GXT, a divergence from the strong linear and subtle curvilinear relationships reported in prior work that used a treadmill GXT protocol with elite male soccer athletes (Batterson et al., 2023; Batterson et al., 2024). Lastly, while longitudinal trends in physiological thresholds are apparent, no statistically significant changes were found for either the LTs or SmO_2_BPs monitored in this cohort throughout the season, which may be partially related to the change in training intensity and volume, size of the cohort monitored, nutritional factors such as energy availability, or other variables. Exploratorily, average GXT SmO_2_resat (mean SmO_2_resat) did display a significant reduction from Nov-Feb following winter break (p = 0.04), with a nearly significant improvement preceding it from Sept-Nov (p = 0.105) which is speculated to be reflective of short-to-moderate term changes in local metabolic O_2_ exchange (i.e., m $\dot{Q}$ O_2_ and m $\dot{V}$ O_2_ balance) (Barstow, 2019) and microcapillary blood volume reperfusion rates (Keller et al., 2023) within the skeletal muscle tissue of interest, both of which may reflect an improved ability to sustain repeated bouts of exercise.

## Data Availability

The raw data supporting the conclusions of this article will be made available by the authors upon reasonable request, without undue reservation.
